# Outcomes in Juvenile-Onset Spondyloarthritis

**DOI:** 10.3389/fmed.2021.680916

**Published:** 2021-05-28

**Authors:** Judith A. Smith, Ruben Burgos-Vargas

**Affiliations:** ^1^Department of Pediatrics, University of Wisconsin School of Medicine and Public Health, Madison, WI, United States; ^2^Departamento de Reumatologia, Hospital General de Mexico, Mexico City, Mexico

**Keywords:** juvenile spondyloarthritis, enthesitis-related arthritis (ERA), disease manifestations and outcomes, prognosis, TNF inhibitor, sacroiliitis

## Abstract

Some studies have suggested children with juvenile onset spondyloarthritis (JoSpA) have a relatively poor outcome compared to other juvenile idiopathic arthritis (JIA) categories, in regards to functional status and failure to attain remission. Thus, in the interest of earlier recognition and risk stratification, awareness of the unique characteristics of this group is critical. Herein, we review the clinical burden of disease, prognostic indicators and outcomes in JoSpA. Of note, although children exhibit less axial disease at onset compared to adults with spondyloarthritis (SpA), 34–62% have magnetic resonance imaging (MRI) evidence for active inflammation in the absence of reported back pain. Furthermore, some studies have reported that more than half of children with “enthesitis related arthritis” (ERA) develop axial disease within 5 years of diagnosis. Axial disease, and more specifically sacroiliitis, portends continued active disease. The advent of TNF inhibitors has promised to be a “game changer,” given their relatively high efficacy for enthesitis and axial disease. However, the real world experience in various cohorts since the introduction of more widespread TNF inhibitor usage, in which greater than a third still have persistently active disease, suggests there is still work to be done in developing new therapies and improving the outlook for JoSpA.

## Introduction

As a whole, the group of children with JoSpA/ERA have worse reported outcomes than other categories of juvenile idiopathic arthritis (JIA) in regards to remission rates, pain, and quality of life (1, 2). Some of the challenges for this group derive from treatment-refractory complications and insidious, sometimes asymptomatic axial disease progression in JoSpA/ERA. The long lag between symptom onset and diagnosis remains problematic as well (3, 4). Greater awareness of the unique clinical attributes in JoSpA/ERA could aid providers in stratifying their patients toward more aggressive therapy. It is also important to identify unmet clinical needs regarding outcomes. Although the therapeutic options have changed over time with the advent of biologics, the real-world impact on outcome is not clear. Thus, the goals of this review are to highlight the clinical characteristics of this group that contribute to the burden of disease, prognostic indicators, and the remaining gaps in outcomes.

In adults, axial SpA encompasses a spectrum of symptoms including pain and stiffness affecting the spinal and sacroiliac joints and axial entheses, and more rarely peripheral arthritis and enthesitis. Familial aggregation (genetics) and significant association with HLA-B27 antigen play an important role in the pathogenesis of the disease (5–7). Besides musculoskeletal involvement, adults with SpA have a variable percentage of anterior uveitis, psoriasis, and gut disease (8). Children and adolescents, by definition those with disease onset ≤ to 18 years of age, present with clinical disease that clearly overlaps with this adult spectrum, although with some differences, likely reflecting the developing immune system, mechanical differences and potentially the microbiome (9, 10). However, because of some of these key clinical differences, the adult classification criteria, particularly those for axial SpA, may not perform particularly well in capturing children. For instance, inflammatory back pain (by definition, pain for more than 3 months), which serves as an entry point for adult disease, is much less common in children early in their disease course (11–13). Related to the lower frequency of sacroiliitis at disease onset in juvenile Spondyloarthritis (JoSpA), one group has reported a sensitivity of only 25% for ASAS axial SpA criteria (14). The ASAS peripheral SpA critera may perform better in children, capturing >90% of subjects (15–17). Unfortunately, there is currently no official JoSpA classification equivalent. The current SpA monikers and classification criteria applied to children is a muddle, including a pot-pourri of terms such as “seronegative enthesopathy and arthropathy” (SEA syndrome), “enthesitis related arthropathy” (ERA), and worse yet, “undifferentiated arthritis” (18). The problems surrounding nomenclature are described in detail in chapter 1 of this issue and so will not be addressed further here. In this chapter, we will generally use the inclusive acronym JoSpA/ERA (juvenile onset spondyloarthritis/ERA), unless specific International League Against Rheumatism (ILAR) classification categories (e.g., ERA, PsA, undifferentiated arthritis) are being described Petty et al. (19).

## Axial and Peripheral Arthritis and Enthesitis in JoSpA/ERA

Compared with other types of JIA, children with JoSpA/ERA have a higher male representation and older age of onset (typically 10–11 years) (basic clinical characteristics in Table 1) (18, 20, 25, 29, 30). HLA-B27 positivity in various JoSpA/ERA cohorts and case series is variable, potentially reflecting ethnic differences. For instance HLA-B27 is present in ~6–8% in Europeans, but rare in Africans and in Japanese (<1%) (31). In general, the prevalence of ankylosing spondylitis (AS) and other related SpA conditions strongly associates with HLA-B27 antigen in different populations around the world (32). Although in some populations, for instance in Africans, SpA may associate more with other HLA molecules (e.g., HLA-B14:03) (33). In the multi-national studies cited here, HLA-B27 prevalence ranges from 35 to 97% [see Table 1 and (26)]. Despite this ethnic variability, HLA-B27 still accounts for the greatest known influence on genetic susceptibility to AS and SpA (6, 34, 35), and is overrepresented in children with SpA compared to the general population (30, 36, 37). Moreover, distribution of HLA-B27 disease-associated subtypes in JoAS (juvenile onset ankylosing spondylitis) mirrors the prevalence in subjects with adult onset AS (36).

**Table 1:** JoSpA/ERA clinical features.

**Years (y)**	**Country**	**Cohort/sample size**	**Y f/u**	**SpA #(%) of total cohort**	**Age onset ±SD or (25%, 75%) or (range)**	**% Male**	**% HLA-B27**	**% Spine involved**^**e**^	**% periph. Arthritis**	**% Enthesitis**	**% Eye**	**References**
<1982	Canada	39	1.9	39 (100) SEA^b^	9.8 (2, 16)	90	72	44 exam 28 X-ray SI 15 X-ray TL spine	74	100	15	(18)
1980–1985	Norway	175	15.3	55 (33) ERA	11.1 ± 2.8	65	85	35 X-ray 47 IBP 75 decreased mobility	–	–	–	(2)
1997–2000	Nordic	410	8	46 (11.2) ERA 14 (3.4) PsA 63 (15.4) UA	10.5 (8.6, 12.3) 5.9 (3.2, 7.2) 8.1 (3.5, 11.9)	65.2 50 28.6	72 21 21	–	–	–	–	(20, 21)
<2001	England	246	28	32 (13.1) ERA 15 (5.3) PsA	10.0 ± 3.3 9.9 ± 3.3	–	–	–	–	–	28 23	(22)
2002–2003	Germany	118	4	118 (100) SpA (mNY or ESSG)^b^	–	73	66	32 IBP	96	44	6.8	(17)
1994–2006	India	235	>1.5	84 (36) ERA 3 (1) PsA 11 (5) UA	13 (7, 16) 12 (5, 12) 11 (2–15.5)	91 33 55	89^d^ – 40	19 SI, 37 spine 0 18 SI, 20 spine	–	37^f^ 0 27	8.3 0 0	(23)
2000–2006	Italy	59	3	59 (100) ERA	9.3 (6.5–13.3)	68	66	36 IBP or decreased mobility (in 1y)	–	–	–	(24)
2006–2009	Brazil	253		253 (100) JSpA (ESSG)		86	80	60 IBP	60 lower limb, 20 upper limb	58	25	(4)
1995–2010	Taiwan	195	>1.5	73 (37) ERA 3 (1.5) PsA 11 (5) UA	10.8 (8.9, 12.3) 9.4 (6.2, 10.8) 10.2 (8.7, 15.1)	85 67 20	82 33 0	48 SI or lumbar 33 0	–	–	9.6 0 0	(25)
1989–2012	USA	234	–^a^	234 (100) ERA	–	72	59	26 clinical SI 56 MRI	92	75	5.6	(26)
2008–2015	France	114	2.5	ERA/JSpA^c^	9.6 (6.9, 12.3)	59	43	63 (47 with SI, 24 thoracic and 44 lumbar)	87	86	–	(16)
1993–2018	Taiwan	181	7.7	72 (40) ERA	11.0 ± 3.2	86	97	16 (clinical or X-ray SI)	–	–	10	(27)

*Cohorts/series are listed by years of patient recruitment and nationality in left columns. Unless otherwise indicated, features are cumulative at follow up rather than baseline. ILAR, International League against Rheumatism; ERA, enthesitis related arthritis; PsA, psoriatic arthritis; UA, undifferentiated arthritis. Follow up years refers to mean or median, depending upon the study. For cohorts not describing a specific manifestation (no data) or with insufficient data (based on <1%), the missing data are designated with a dash (–). IBP, inflammatory back pain; SI. sacroiliitis*.

a *Cross-sectional study*.

b *Other JoSpA. SEA, seronegative enthesopathy and arthropathy; ESSG, European Spondyloarthropathy Study Group; Mny, modified New York criteria for ankylosing spondylitis (28)*.

c *Physican diagnosed JSpA. At last followup, 92% met clinical criteria for ASAS peripheral SpA and 75% for either ERA or PsA*.

d *Percentages are from 62 patients with ERA and 10 with PsA were tested for HLA-B27*.

e *Description of axial involvement was heterogeneous between sources and included symptoms, clinical assessment, radiology (X-ray), and MRI*.

f *Relatively low proportions of enthesitis in “ERA” are not explained*.

In comparison with adult onset AS, children with JoAS tend to present with more enthesitis and peripheral arthritis and less lumbar pain and stiffness, a pattern also characteristic of the greater spectrum of JoSpA/ERA (4, 11, 38). Presence of enthesitis varies by study location and definition of SpA, ranging from 37% in ERA to >100% in SEA (Table 1, with specific anatomic distribution described in Table 2) (18, 23). The most frequent areas of involvement are the calcaneal insertion of the Achilles tendon, patellar tendons and insertions of the plantar fascia. Clinical assessment of enthesitis can be challenging; however, ultrasound has been an extremely useful, though operator-dependent adjunct, as has MRI (41, 42). Interestingly enthesitis is not exclusive to ERA. One of the largest studies to date on the topic of enthesitis in different JIA categories comes from Rumsey et al. (43). In a Canadian JIA inception cohort, enthesitis was defined by entheseal tenderness in more than one body site on more than one occasion during 60 months follow up. Enthesitis affected 16% of this large JIA cohort (1,406 patients), and ERA, PsA and undifferentiated arthritis accounted for 64, 2, and 18%, respectively, of those with enthesitis. In this cohort, children with enthesitis tended to be older at disease onset (10.7 vs. 7.5), male (57 vs. 31%), have polyarthritis (57 vs. 41%), and sacroiliitis (30 vs. 4%). Within ERA, 141/202 (70%) had enthesitis. In this JIA cohort, the most common anatomical locations were the plantar fascia (39%), Achilles (31%), and tibial tuberosity (30%). The course of enthesitis tended to follow active joint count (43).

**Table 2:** Anatomic distribution of peripheral arthritis and enthesitis.

**Years (y) recruited**	**<1982**	**1980–1985**	**2002–2003**	**2000–2006**	**1995–2010**	**1989–2012**	**2008–2015**	**2015–2016**
**Country**	**Canada**	**Mexico**	**Germany**	**Italy**	**Taiwan**	**USA**	**France**	**India**
Cohort size	39	110	118	59	195	234	114	55
Y f/u	1.9	12.2	4	3	>1.5	–^b^	2.5	<5
SpA # (%)	All SEA	35(32) JoAS	All SpA mNY or ESSG	All ERA	73(37) ERA	All ERA	All SpA	All ERA
Peripheral arthritis								
% Knee	83	100	77	65	52	46	58	–
% Ankle	–	80	40	48	38	36	38	27
% Hip	–	83	38	–	43	19	46	–
% Mid-foot	–	89	9.3	58	–	–	9	36 by ultrasound 54 by MRI
% Fingers	–	23	25	–	18^a^	–	12	–
% Toes or MTP	–	86	27	–	16^a^	–	17	4 toes 16 MTP
% Wrist	–	14	–	–	16	20	25	–
% Dactylitis	–	–	13	–	–	–	13	7.3 (toes)
Enthesitis			–		–			
% Achilles	51	34		28		33	74^c^	44
% Plantar front insertion	–	–		–		–	39	20^d^ See note
% Plantar calcaneal insertion	67	54		38		–	See note^c^	See note^d^
% Knee	49	23		–		44	46	–
% Pelvis	5	9		–		30	22^c^	–
% Greater trochanter	–	14		–		–	See Note^c^	–
**References**	(18)	(39)	(17)	(24)	(25)	(26)	(16)	(40)

*Cohorts are listed across the top by years of recruitment and nationality. ERA, enthesitis related arthritis; ESSG, European Spondyloarthropathy Study Group; mNY, modified New York criteria for ankylosing spondylitis (28). ASAS, Assessment of Spondyloarthritis International Society; F/u, median or mean follow up, depending upon the study; MTP, metatarsal-phalangeal joints. No data or insufficient data (<1%) designated as –. Knee includes tibial tuberosity, infrapatellar, and suprapatellar sites. Pelvis includes ischial tuberosity, iliac crest, and interosseous ligaments of sacroiliac joint*.

a *Specified as “small joints” of fingers or feet*.

b *Cross sectional inception cohort without specified follow up*.

c *74% had Achilles or plantar calcaneal insertion enthesitis. “Pelvis” was lumped with greater trochanteric enthesitis in this study*.

d *Not specified if frontal or calcaneal plantar fasciitis*.

Regarding arthritis phenotype, children with JoSpA/ERA typically present with asymmetric oligoarticular arthritis affecting the large weight-bearing joints (knees), ankles, mid-foot, and root joints (hips and shoulders) (17, 18, 26, 39, 44). Table 2 presents the anatomic distribution described in several JoSpA/ERA cohorts and case series. In a long-term study from Norway, 73% had oligoarticular onset, and in US and Taiwanese cohorts, 78 and 97% had oligo articular onset, respectively (2, 26, 27). A few studies have reported >50% prevalence of polyarticular involvement in JoSpA/ERA, and patients can accumulate 5 or more joints over time (17, 21, 39, 43). The most commonly affected joints include the knees (46–100%) and ankles/subtalar joints (27–80%). Hip arthritis is also common (19–83%, Table 2) and can be relatively aggressive and severe (45). Indeed, 2 studies comparing juvenile and adult onset AS described increased rates of hip arthroplasty in the JoAS group (17.7 vs. 8.7% in the Genseler study and 17 vs. 4% in the Calin study), although no difference was reported in a Canadian study (7% for both, O'Shea et al.) (38, 45, 46).

Foot arthritis, particularly mid-foot arthritis or tarsitis, is highly characteristic of this population, though the prevalence varies depending upon the study (Table 2). Indeed, in one study comparing JoAS and other Juvenile Rheumatoid Arthritis (JRA), 85.7% of children with JoAS experienced tarsitis, vs. 10.7% in JRA within 1 year of presentation (39). Anatomic involvement of the feet was particularly well described in a study from India (40). Phatak et al. described a case series of 55 children diagnosed with ERA for <60 months. This population was 96% male and 80% HLA-B27 positive, with sex most likely skewed by referral bias. This sex bias may over-represent the incidence of this complication in JSpA but anatomic distribution should still be generally informative. Foot pain occurred at presentation in 56% and another 27% developed foot pain during <5y follow up. Foot/ankle arthritis was apparent by exam in 65%, with talo-Achilles enthesitis in 47% and plantar fasciitis in 20%. Ultrasound was abnormal in 56% and MRI in 54%. Most of the MRIs that were positive in clinically asymptomatic subjects only had bone marrow edema, which may or may not have been pathologic. By exam, the most commonly involved arthritic joints were the midfoot (44%) followed by tibiotalar (27%), subtalar (15%), and MTP (16%). By ultrasound and MRI, the most commonly affected joints were talonavicular and tibiotalar, followed by calcaneocuboid and subtalar. Ankle arthritis prevalence was 27%. In those with mid-foot disease, half had mid-foot enthesitis and 2/3 tenosynovitis. Foot involvement correlated with significant functional impact, based on their juvenile arthritis foot index (JAFI). None of the children received biologics. There was no correlation with sacroiliitis or HLA-B27. Similar early prevalence was described in a Spanish cohort, where 35% had tarsitis at presentation (47). In this series, the children with tarsitis were much less likely to present with axial pain (8 vs. 54%) or develop axial involvement, and were often initially misdiagnosed with infection. These results contrast with those obtained by Burgos-Vargas et al., in which tarsitis (in conjunction with enthesitis) was highly predictive of a diagnosis of AS at 10 years (39).

“Inflammatory Back Pain” seldom occurs in children. In addition, some of the metrics used to follow adults are not helpful in children. For instance, in one study, chest expansion was the same for children with JoAS and SEA and healthy children (48). Moreover, there is poor correlation between symptoms, exam findings and disease indicated by MRI, making clinical assessment challenging (49). However, it is important to maintain a high index of suspicion during initial assessment and to remain vigilant for the development of axial disease. A significant portion of children with JoSpA/ERA have axial involvement at presentation or develop axial disease within the first 5 years, with some variability by study. For example, in the predominantly male and HLA-B27+ ERA case series from India, 55% had inflammatory back pain (IBP) and 38% clinical sacroiliitis during the first 5 years of disease (mean duration of disease 1.9 years) (40). Of these, 33% had radiographic sacroiliitis. In one of the largest multinational MRI-imaged inception cohort of 540 children with clinically suspected JoSpA, 20% had sacroiliitis. Interestingly 42% had incidental findings unrelated to the sacroiliac joints that potentially contributed to axial symptoms, including enthesitis, hip arthritis, and degenerative disease (50). Even in cohorts where sacroiliitis incidence was initially <30%, more than half of the subjects developed axial disease within 5 years (16). In a 1989 report, in an SEA cohort of 20 children, 47% fulfilled modified NY criteria for ankylosing spondylitis (evidence by X-ray) within 3 years, 75% in 5 years and >90% in long-term follow up (51). In another study of children with JoAS, only 14% had lumber or sacroiliac pain 1 year after disease onset, although 100% reported sacroiliac/lumbar symptoms by 10 years (39).

Recent MRI-imaging studies of children with JoSpA/ERA have revealed an alarming percentage with asymptomatic axial disease. In a SpA cohort reported by Weiss at al. 20% had MRI-detected active sacroiliac disease at presentation. A high proportion (88%) of these cases already exhibited erosions, but only 38% of those with positive MRIs reported any back symptoms (49). In another cohort of 143 JoSpA patients, 53 (37%) of the patients had imaging or clinical suggestions of axial involvement. Eighteen had abnormal sacroiliac X-rays and 20 had sacroiliitis by MRI (32 total with abnormal imaging), but a third of these (11) had no back symptoms (52). Given the prevalence of axial inflammation at baseline, propensity for developing axial disease, and occurrence of axial disease in the absence of reported back pain in some children, there should be a low threshold for evaluating children with JoSpA/ERA by MRI. In adults with AS, early disease is a time-limited opportunity to gain the greatest response from biologic medication such as TNF inhibitors (53, 54). Unfortunately lag between symptom onset and diagnosis is even longer in children than in adults (8–9 vs. 5 years) (3, 55).

## Extra-Articular Manifestations

Depending upon the study and length of follow up, uveitis has been reported in 5–28% of JoSpA/ERA subjects [(22, 26), Table 1]. A comprehensive cross-sectional/retrospective study came out recently describing uveitis among 118 children with JoSpA, including ERA (62% of the SpA cases), PsA (18% of the cases), undifferentiated arthritis (14%), and IBD-associated arthritis (6%). Uveitis was reported in 24 subjects (11%), with the highest proportion in ERA (13% of those patients) and 7% in the other ILAR SpA categories. Seventy nine percent of the uveitis was symptomatic. HLA-B27 prevalence, at 45%, was similar between groups and did not correlate with likelihood of uveitis, nor with symptomatic uveitis (56).

Skin and nail involvement characterizes the PsA ILAR subgroup. Clinical manifestations for this subgroup have been reviewed extensively elsewhere and will not be described in detail here (57–59). Briefly, children with nail involvement may exhibit nail pitting and onycholysis. Children do not always manifest the pathognomonic discrete erythematous scaly plaques. Psoriasis may be subtle and confused with eczema in children. Places that may exhibit scaling are along the hairline, behind the ears, around the umbilicus and intergluteal cleft. Dactylitis, or diffusely swollen “sausage” digits are also common in children with PsA. Prevalence in JSpA overall is ~10% (Table 2) (16, 17, 40).

In adults, between 6 and 14% of patients with AS develop frank IBD (60). However, colonoscopies from asymptomatic or mildly symptomatic patients have revealed a shocking prevalence of subclinical inflammation in ~60% of subjects (61). These findings and other studies have suggested the potential involvement of a gut-joint axis in disease pathogenesis (62, 63). In children, gut involvement in JoSpA/ERA is less clear, not the least because of the difficulties in classification. However, one study found 67% of children with ERA had elevated calprotectin compared to 18% in other types of JIA, supporting the concept of a disease continuum between childhood and adult onset SpA (15, 64).

Heart disease is certainly less common in children with AS vs. adults, though a 1995 study in a 36 patients with JoAS revealed 2 patients with mild mitral regurgitation and 3 with aortic regurgitation (65). None had functional impact or conduction abnormalities. In the initial description of SEA, 2 of 39 subjects (5%) had aortic insufficiency (18). By way of comparison, 5–10% of adults with AS have a conduction disorder or aortic insufficiency (66). Although functional cardiac complications may be relatively infrequent in JoSpA/ERA, these studies suggests the heart might be an organ worth monitoring in children. At the very least, more data on this topic would be helpful.

## Prognostic Indicators and Outcomes

Various studies, particularly those from before the biologic era, paint a relatively dismal prognosis for children with SpA (Table 3), particularly when considering disability, pain, and remission rates. In a long-term Norwegian cohort, at 15 years patients had a lower level of physical function indicated by HAQ scores (0.38 vs. 0.16) and poorer physical health (SF-36, 46.4, vs. 52.4) and pain (2.88 vs. 2.09 on 1-6 scale) vs. polyarticular and oligoarticular JIA (2). In the 30-year follow up of that same cohort comparing ILAR subgroups, only 37% of ERA patients were in remission off medications, and the only group that fared worse than ERA was RF+ poly JIA (67). Similarly, in a large cross-sectional study using CARRA registry data, Weiss et al. reported that children with ERA had worse pain, function (CHAQ) and health status than other forms of JIA (1). In the Canadian ReACCh-Out JIA cohort, a diagnosis of ERA carried an OR of 0.67 and undifferentiated arthritis an OR of 0.49 for attaining inactive disease (72).

**Table 3:** Remission vs active disease in Juvenile Spondyloarthritis cohorts.

**Years recruited**	**Country**	**Y f/u**	**SpA #(%) of total cohort**	**% Active**	**% Remission (total)**	**% Remission (on med)**	**% Remission (off med)**	**% Continuous active**	**% Remit for 1y then flare**	**% TNFi**	**References**
1980–1985	Norway	15.3	55(33) ERA	56	44	–	–	–	–	0	(2)
1980–1985	Norway	30	27(15) ERA 21(12) PsA 11(6) UA	–	–	–	37 48 64	–	–	0	(67)
1970–1998	Italy	10	67 (10) SpA (ILAR/ESSG)	64	36	–	–	52	15	–	(68)
1997–2000	Nordic	7	49(11) ERA 14(3.2) PsA 66(15) UA	61 54 53	39 46 47	8 23 6	31 23 41	31 27 40	–	17.5 for all JIA	(20)
2002–2003	Germany	4	118 SpA (mNY or ESSG)	54^a^	–	43^a^	23^a^	–	14	6	(17)
1995–2010	Taiwan	>1.5	73(37) ERA 3(1.5) PsA 5(2.6)UA	56 0 20	44 100 80	11 33 0	33 67 80	48 0 0	8 0 20	12.8 for all JIA	(25)
2005–2010	Canada	5	157(14) ERA 64(6) PsA 110(10) UA	53 53 54	47 47 46	–	–	–	–	<20	(69)
2005–2010	Canada (2 of above centers)	5.6	52(21) ERA 10(4) PsA 13(5) US	35 20 15	65 80 69	13 30 15	52 50 54	–	–	22 for all JIA	(70)
2013–2014	Germany	1	74(11) ERA 28(4) PsA 50(7) UA	72 73 58	28^b^ 29 42	–	–	–	–	25 32 36	(71)
2008–2015	France	2.6	114 ERA or ASAS	45	55	35	20	–	–	42	(16)
1993–2018	Taiwan	7.7	73(40) ERA 1(0.5) PsA 1(0.5) UA	66	34	7	27	–	–	78	(27)

*Cohorts are listed by years of patient recruitment and cohort nationality (left columns)*.

*Cohort acronyms: GESPIC, German Spondyloarthritis Inception Cohort; ReACCh-Out, Research in Arthritis in Canadian Children Emphasizing Outcomes); ICON, Inception Cohort of Newly Diagnosed Children with JIA; JCA, juvenile chronic arthritis; JRA, juvenile rheumatoid arthritis; JIA, juvenile idiopathic arthritis; ERA, enthesitis related arthritis; PsA, psoriatic arthritis; UA, undifferentiated arthritis; ESSG, European Spondyloarthropathy Study Group; mNY, modified New York criteria for ankylosing spondylitis; ILAR, International League against Rheumatism; ERA, enthesitis related arthritis; PsA, psoriatic arthritis; UA, undifferentiated arthritis; ASAS, Assessment of Spondyloarthritis International Society. Follow up years refers to mean or median, depending upon the study. Cohort specific details are in footnotes. TNFi: TNF inhibitor usage by end of study*.

a *54% in active disease after 4y. 43% in remission on meds and 23% in remission off meds at 4y or within past 6 months*.

b *Status of active or inactive disease during months 9–12. In ERA, 55% eventually attained disease remission, but at a mean of ~16 months*.

Multiple studies point to baseline enthesitis as a poor prognostic indicator, despite its responsiveness to current therapeutic approaches (1, 43, 71, 73). One possible explanation for this association is that enthesitis often portends sacroiliitis (16). Indeed, several studies have directly implicated sacroiliitis as a poor prognostic indicator. In a Taiwanese JIA cohort followed over 8 years, any sign of sacroiliitis (clinical or radiologic) predicted active disease, as none of these subjects attained inactive disease (27). In the Berntson cohort, any signs of sacroiliitis (OR 4.1), enthesitis (OR 2.4) or hip arthritis (OR 2.1) increased the risk for persistent disease. For sacroiliitis alone, 84% exhibited active disease (21). Herregods et al. noted the association between enthesitis and sacroiliitis in an imaging study, where sacroiliitis was present in 74% of children with pelvic enthesitis (74). In an Italian MRI study in ERA, early predictors of sacroiliac disease were numbers of active joints and active entheses at onset (24). Earlier studies had also reported an association between high joint count and ultimate development of sacroiliitis; in a study comparing initial patterns of arthritis (pauci vs. poly) between children with JoAS and those with SEA who did not develop axial disease, polyarticular disease at 1 year was highly associated with the development of radiographic sacroiliitis/AS (75). In another study examining joint accumulation over time in a group of children ultimately diagnosed with JoAS, at 6 months after disease onset, only 28.6% of patients had polyarticular disease, but by 1 year, that number increased to 80% (39). In a Norwegian ERA cohort, high active joint count at 6 months predicted physical limitation at 23 years (2). Besides high joint count and enthesitis, other risk factors for the development of sacroiliitis include family history of SpA, persistently high ESR and hip arthritis (2, 16, 21, 24, 76).

Several studies have examined the influence of HLA-B27 positivity, sex, and their interaction on the presence of sacroiliitis and more directly on prognosis. In a study from Berntson et al. focusing on HLA-B27 across JIA categories in a Nordic cohort, HLA-B27 positivity associated with clinical signs of sacroiliitis (including inflammatory back pain, sacroiliac and buttock pain), enthesitis, and tenosynovitis in boys, but not girls (21). In the whole JIA cohort, boys were more often HLA-B27+ (26%) vs. 18% of girls, and boys with ERA had a trend toward clinical signs of sacroiliitis more often than girls (21). In a Norwegian ERA cohort followed over 15 years, male sex also associated with the development of decreased spinal mobility (abnormal Schober test) in 67% of 36 boys vs. 37% of 19 girls) (2). However, in a French JoSpA/ERA cohort followed over 5 years, axial disease and sacroiliitis prevalence was equally distributed between sexes (16). In several studies from Taiwan, Germany and Norway, HLA-B27 positivity associated with failure to attain remission (17, 21, 25, 27). The relative odds ratios were around 2 (1.7–2.2), correlating with persistence of active disease after 8 years of 73.2 vs. 59.4% in the whole JIA cohort (21, 25). Interestingly, in the GESPIC and Norwegain cohorts, female sex carried a worse prognosis (2, 17). Thus, there have been some cohort or analysis-specific differences regarding sex.

## Remission Rates and Biologic DMARDS

One possible reason for the relatively poor outcomes noted in various studies, is that the most prominent aspects of JoSpA/ERA (enthesitis and sacroiliitis) do not respond well to conventional synthetic disease modifying anti-rheumatic drugs (DMARDs) such as methotrexate or sulfasalazine (reviewed in another chapter in this issue) (77, 78). The data supporting these contentions, mostly obtained from adult studies, has led to revisions in current ACR treatment guidelines supporting earlier use of biologic DMARDs (e.g., TNF inhibitors) following NSAID failure in children with sacroiliitis or enthesitis (79). The IL-17 inhibiting monoclonal antibodies are too new to assess in children, however TNF inhibitors have become much more widespread in use in the 2000s. Trial data has been promising (reviewed in another chapter), but cohorts indicate “real world” application, and how outcomes may be shifting (or not). For simplicity, we will focus on active disease vs. remission over time (Table 3). Over time, there has been a steady increase in biologic DMARD usage, yet a corresponding increase in remission vs. persistent active disease is not yet clear.

A few studies suggest the outlook for ERA may be improving (Table 3), particularly in the short term. For instance, in an open-label study of JSpA treated with TNF inhibitors, 81% (13/16) achieved clinical remission within 6 months. However, 6/16 (38%) subsequently flared a median of 2 years after attaining remission (80). In a French study, 69% of subjects treated with TNF inhibitors experienced inactive disease at 1y, with boys exhibiting a greater response (OR 6.94) (16). Experience in a Canadian cohort (ReACCh-Out) also suggested good short-term gains in SpA; the probability of attaining inactive disease some time during 5y follow up was extremely high, at >92% for all SpA categories. Probability of coming off meds during the 5y was 71% for ERA, 74% for PsA and 59% for undifferentiated arthritis. However, overall remission rates at the end of the study were still <50% (69). A subset of this same cohort examined a few years later achieved remissions >60% (20–35% with active disease), marking an improvement in outcome, though this may be specific to the two centers examined (70). In a German etanercept cohort, only 52% of ERA patients attained inactive disease, with 22% in remission on medication (81). Comparable outcomes were reported in a 2020 comprehensive study on unmet needs in JIA. Brunner et al. described 2 large cohorts, one from Cincinnati Children's (CHMC) and another from the multi-site Childhood Arthritis and Rheumatology Research Alliance (CARRA) registry, including 279/1351 with ERA/PsA and 50/164 with undifferentiated arthritis (82). In the two cohorts, 79 and 72% of children with ERA/PsA were treated with biologics. Even among those treated with two biologics, 31% (CHMC) and 55% (CARRA) still had active disease. While these outcomes are much better than those reported by Minden et al. in 2000 [17% remission at 5y (83)], there is still obviously room for improvement.

Children with JoSpA/ERA treated with TNF inhibitors may still experience a worse outcome compared to other types of JIA. In the German ICON cohort, even though 30% of ERA patients were treated with biologic DMARDs, this group took longer than other categories of JIA to attain a state of inactive disease (9m) and spent only 27% of time in inactive disease within the first year (compared to 40% in the whole JIA cohort). PsA patients spent 25% in inactive disease. Only 55% of ERA patients reached inactive disease at a mean of 15.9m, leaving 45% with active disease more than a year from enrollment (71). In a study from Taiwan, only 33% achieved inactive disease, despite high levels of treatment with TNF inhibitors (78%), an outcome that was still significantly worse than for other JIA groups (27). Even within a JIA cohort started on TNF blockers, patients with ERA were much less likely than those with poly JIA (RF-) to attain inactive disease ever (43 vs. 76%) or be in inactive disease at 1y (24 vs. 57%) (73). Part of the issue may reflect TNFi refractory disease manifestations typical of JoSpA/ERA. For instance, the aggressive hip arthritis may be resistant to TNFi therapy (80). Similarly, TNFi may have limited capacity to suppress progression of sacroiliitis. In the 2014 open label study of etanercept and infliximab, 42% of children met modified NY AS criteria prior to treatment, and 92% fulfilled criteria 7 years later (80).

In summary, children with JoSpA/ERA have significant disease burdens and relatively poor outcomes compared to other types of JIA (1). Although most patients initially present with peripheral arthritis and enthesitis, a very high proportion go on to develop axial disease, within the first 5 years of diagnosis (11, 51). An alarming number of these children (one third to over one half!) develop “silent” axial disease and have MRI evidence for both acute disease and chronic destructive changes, even in the absence of reported back pain (49, 52). Moving forwards, it will be critical to determine if there are other clinical features that correlate more reliably with axial disease. Another possible solution is to treat children diagnosed with JoSpA more aggressively early on, incorporating TNF inhibition for their peripheral arthritis and enthesitis prior to the development of axial disease. Greater TNF inhibitor use may be improving the outcome in this difficult to treat JIA category, particularly in the short term, though more data would be helpful for supporting this idea. The development of sacroiliitis portends a relatively poor prognosis and increased refractoriness to treatment (27). Limited data also suggests axial disease may progress despite TNF inhibitor treatment (80). Thus, the window of opportunity may be limited. At this time, persistently active disease in 30 to >50% of children indicates that there is still much to accomplish toward improving the outlook for JoSpA/ERA.

## Author Contributions

JS and RB-V conceived the review. JS wrote the first draft of the manuscript. RB-V contributed to revision of the manuscript prior to submission.

## Conflict of Interest

The authors declare that the research was conducted in the absence of any commercial or financial relationships that could be construed as a potential conflict of interest.
